# Semi-automated Rasch analysis with differential item functioning

**DOI:** 10.3758/s13428-022-01947-9

**Published:** 2022-09-07

**Authors:** Feri Wijayanto, Ioan Gabriel Bucur, Karlien Mul, Perry Groot, Baziel G.M. van Engelen, Tom Heskes

**Affiliations:** 1 Institute for Computing and Information Sciences, Radboud University Nijmegen, Nijmegen, The Netherlands; 2 Department of Informatics, Universitas Islam Indonesia, Yogyakarta, Indonesia; 3 Department of Neurology, Donders Institute for Brain, Cognition, and Behaviour, Nijmegen, The Netherlands

**Keywords:** Semi-automated Rasch analysis, Rasch model, Generalized partial credit model, Penalized JMLE, GPCMlasso, GPCM-DIF, Differential item functioning, DIF detection

## Abstract

Rasch analysis is a procedure to develop and validate instruments that aim to measure a person’s traits. However, manual Rasch analysis is a complex and time-consuming task, even more so when the possibility of differential item functioning (DIF) is taken into consideration. Furthermore, manual Rasch analysis by construction relies on a modeler’s subjective choices. As an alternative approach, we introduce a semi-automated procedure that is based on the optimization of a new criterion, called in-plus-out-of-questionnaire log likelihood with differential item functioning (IPOQ-LL-DIF), which extends our previous criterion. We illustrate our procedure on artificially generated data as well as on several real-world datasets containing potential DIF items. On these real-world datasets, our procedure found instruments with similar clinimetric properties as those suggested by experts through manual analyses.

## Introduction

In measurement theory, personal aspects may contain latent constructs or traits which cannot be approached directly, such as “intelligence” and “quality of life”. In an effort to measure these latent constructs, many scales have been developed from uniquely designed questionnaires. Rasch analysis is one of the scientific methods to transform the original survey into a linear-weighted, clinimetrically sound scale. Using inherent criteria, e.g., goodness-of-fit, unidimensionality, and local dependency (Mesbah, 2010), manual Rasch analysis follows a step-by-step procedure, repeatedly fitting the observed responses to the Rasch model. The worst item(s) are generally removed, after which the remaining items are reevaluated, until a clinimetrically optimal itemset has been obtained.

Rasch analysis becomes even more complex when the original survey contains items that can function differently due to the respondents’ backgrounds (e.g., age, gender, and nationality). This phenomenon is known as differential item functioning (DIF) (Holland & Wainer, 1993). DIF occurs if respondents from a particular group tend to score higher or lower on a particular item compared to other group(s), despite having otherwise similar characteristics. This type of item is often found in clinical observations, for example in running, old people tend to have more trouble compared to young people. Erroneous ignorance of such biases leads to a biased instrument (Borsboom, 2006; Kopf, Zeileis, & Strobl, 2015). DIF assessment has become one of the standard ingredients of Rasch analysis and has been implemented in various ways, e.g., (Holland & Thayer, 1986; Swaminathan & Rogers, 1990; Kreiner & Christensen, 2011; Magis & Facon, 2013; Tutz & Schauberger, 2015; Komboz, Strobl, & Zeileis, 2018; Schauberger & Mair, 2020; Schneider, Strobl, Zeileis, & Debelak, 2021).

In current practice, step-by-step procedures are carried out manually by the experts, which can be relatively time-consuming even with the support from the available software packages, such as (Choi, Gibbons, & Crane, 2011; www.rasch.org, 2014; Magis & Facon, 2014; Jeon & Rijmen, 2016; Bollmann, Berger, & Tutz, 2018). Decisions on how to prioritize the various evaluation criteria and which items to include partly rely on human judgments blended with clinical expertise, and different experts may obtain different but equally suitable instruments. These procedures will become even more complex when the DIF items have to be resolved iteratively (Andrich & Hagquist, 2015; Hagquist & Andrich, 2017).

The objective of this research is to incorporate the DIF assessment procedure while automating the Rasch analysis. In doing so, we extend our previous method, which automates the Rasch analysis using the in-plus-out-of-questionnaire log likelihood (IPOQ-LL) criterion (Wijayanto, Mul, Groot, van Engelen, & Heskes, 2021). The extended method naturally incorporates standard Rasch criteria, e.g., item goodness-of-fit and unidimensionality (Wijayanto et al., 2021). Additionally, we expect the method to perform fairly well, automatically, even though it does not address local dependencies directly: reliable estimation of abilities fares better from items with uncorrelated residuals than those with correlated residuals (Wijayanto et al., 2021). Accordingly, we will show that our new procedure in addition naturally incorporates the standard DIF assessment in Rasch analysis. Our novel procedure makes use of a generalization of the IPOQ-LL criterion, which we will refer to as the in-plus-out-of-questionnaire log likelihood with DIF (IPOQ-LL-DIF).

The rest of this article is structured as follows. “Preliminary” section describes the central model in our implementation, the GPCMlasso model (Schauberger & Mair, 2020), its transformation to other models, and the idea to solve its estimation problem using the *L*1 (lasso) penalty together with the coordinate descent. “The proposed method” section discusses the main part of our proposed method, the in-plus-out-of questionnaire log likelihood with DIF (IPOQ-LL-DIF), which extends the previous method and argues for the method in comparison with the typical assessment of DIF items in standard Rasch analysis. “Experimental study” section reports our experimental results on an artificial and three real-world datasets. “Discussion and conclusions” section discusses general aspects of our procedure and the results it obtained, and concludes our research. The R package containing the algorithm and results reported in this paper can be found at https://github.com/fwijayanto/autoRasch.

## Preliminary

### Generalized partial credit model with DIF

Differential item functioning (DIF) refers to the situation where members from different groups (age, gender, race, education, culture) on the same level of the latent trait (disease severity, quality of life) have a different probability of giving a certain response to a particular item (Chen & Revicki, 2014). In short, DIF occurs as a result of an inconsistency between estimated abilities and true abilities for given groups. If the inconsistency uniformly affects all subjects in the group, then it is known as a uniform DIF, otherwise, it is a non-uniform DIF (Hagquist & Andrich, 2017). Additionally, Penfield (2007) discusses the complexity of the DIF in the polytomous case by introducing differential step functioning (DSF), which allows an item not only to have differential functioning at the item level but also at the category level. DSF simplifies to DIF when there is a constant difference between groups at the category level. For now, we consider the DIF and provide more details on DSF in Appendix 2.

In this work, we focus on uniform DIF and adopt the GPCMlasso model, introduced in Schauberger and Mair (2020), which extends the generalized partial credit model (GPCM) (Muraki, 1992) after parameterizing the DIF effects. Rooted to the GPCM, the GPCMlasso has the ability to model responses that are coded into two or more ordered categories. We write *x*_*ni*_ ∈{0,1,…,*m*_*i*_} for the observed response of subject *n* on item *i*, where item *i* consists of *m*_*i*_ + 1 ordered categories. We have *m*_*i*_ = 1 for dichotomous test items and *m*_*i*_ > 1 for polytomous items.

The GPCMlasso model contains the same type of parameters as the GPCM: *𝜃*_*n*_ for the *ability* of subject *n*, *β*_*ij*_, with *j* = 1,…,*m*_*i*_ for the *difficulties* or *thresholds* of item *i*, and *α*_*i*_ for the *discrimination parameter* of item *i*. Additionally, to model the difference in difficulty on item *i* between the members and non-members of focal group *f*, the *DIF parametersδ*_*if*_ are introduced. Furthermore, *κ*_*nf*_, with *f* = 1,…,*m*_*f*_ and where *m*_*f*_ represents the number of potential DIF-inducing covariates, is a binary matrix that maps subject *n* into group *f* with *κ*_*nf*_ = 1 if respondent *n* is a member of group *f* and *κ*_*nf*_ = 0 otherwise.

Given these definitions, the probability of subject *n* gives response *x* on item *i* reads

1 $$\begin{array}{@{}rcl@{}} &&P(X_{ni}=x|\theta,\beta,\alpha,\delta)\\ &=& \frac{\displaystyle \exp \left[\alpha_{i} \sum\limits_{j=1}^{x} \left(\theta_{n}-\left(\beta_{ij}-\sum\limits_{f=1}^{m_{f}}\delta_{if}\kappa_{nf}\right)\right)\right]}{\displaystyle 1+\sum\limits_{k=1}^{m_{i}}\exp\left[\alpha_{i} \sum\limits_{j=1}^{k} \left(\theta_{n}-\left(\beta_{ij}-\sum\limits_{f=1}^{m_{f}}\delta_{if}\kappa_{nf}\right)\right)\right]} , \end{array}$$

for *x* > 0, and

2 $$\begin{array}{@{}rcl@{}} &&P(X_{ni}=0|\theta,\beta,\alpha,\delta)\\& =& \frac{\displaystyle 1}{\displaystyle 1+\sum\limits_{k=1}^{m_{i}}\exp\left[\alpha_{i} \sum\limits_{j=1}^{k} \left(\theta_{n}-\left(\beta_{ij}-\sum\limits_{f=1}^{m_{f}}\delta_{if}\kappa_{nf}\right)\right)\right]} . \end{array}$$

From now on, we will refer to this as the generalized partial credit model with differential item functioning, GPCM-DIF. Setting *α*_*i*_ = 1 for $i = 1,\ldots ,\mathbb {P}$ in the GPCM-DIF model gives what we will refer to as the partial credit model with DIF (PCM-DIF). Using the PCM-DIF to estimate the respondents’ traits is comparable to the use of the partial credit model (PCM) on items after the DIF has been resolved. With *δ*_*if*_ = 0 for $i = 1,\ldots ,\mathbb {P}$ and *f* = 1,…,*m*_*f*_ we obtain the GPCM. By then also fixing *α*_*i*_ = 1 for $i = 1,\ldots ,\mathbb {P}$, we get the PCM. In the case of binary responses, with *m*_*i*_ = 1 for $i = 1,\ldots ,\mathbb {P}$, the GPCM transforms to the 2-parameter logistic (2PL) model and the PCM transforms to the original Rasch model (Masters, 1982; Lord & Novick, 1968; Rasch, 1960).

### Coordinate descent

Given observed responses *x*_*ni*_, the log likelihood of all model parameters for a given set of items $\mathcal {S} \subset \{1,\ldots ,\mathbb {P}\}$ reads

3 $$L_{\mathcal{S}}(\theta,\beta,\alpha,\delta) = \sum\limits_{i \in \mathcal{S}} \sum\limits_{n=1}^{N} \log P(X = x_{ni}|\theta,\beta,\alpha,\delta) ,$$

with *P*(*X* = *x*_*ni*_|*𝜃*,*β*,*α*,*δ*) from Eqs. 1 and 2. This log likelihood measures how well the parameters predict the subjects’ observed responses on the items from set $\mathcal {S}$.

We turn the log likelihood into a penalized log likelihood by adding penalty terms. As in Wijayanto, Mul, Groot, van Engelen, and Heskes (2021), we add Tikhonov regularization for the abilities *𝜃*, to regularize these towards zero, as well as for $\ln \alpha$, to drive the discrimination parameters towards one. Inspired by Schauberger and Mair (2020), we further add a Lasso (*L*1) penalty for the DIF parameters *δ*, so that irrelevant DIF parameters are optimized to zero:

4 $$\begin{array}{@{}rcl@{}} F_{\mathcal{S}}(\theta,\beta,\alpha,\delta)& =& L_{\mathcal{S}}(\theta,\beta,\alpha,\delta) - \lambda_{\theta} \sum\limits_{n=1}^{N} {\theta_{n}^{2}}\\&& - \lambda_{\alpha} \sum\limits_{i \in \mathcal{S}} (\ln \alpha_{i})^{2} - \lambda_{\delta} \sum\limits_{i \in \mathcal{S}}\sum\limits_{f=1}^{m_{f}}|\delta_{i f}| \end{array}$$

with *λ*_*𝜃*_, *λ*_*α*_, and *λ*_*δ*_ the penalty coefficients of *𝜃*, *α*, and *δ* parameters, respectively.

To optimize (4), we propose to apply two-level coordinate descent (Friedman, Hastie, Höfling, & Tibshirani, 2007). At the top level, we treat the GPCM parameters *𝜃*, *α*, and *β* as one coordinate, and the DIF parameters *δ* as another. Given fixed DIF parameters, we optimize the GPCM parameters using penalized joint maximum likelihood estimation (PJMLE). As an alternative, we could here replace the PJMLE by marginal maximum likelihood estimation (MMLE), which optimizes the *β* parameters after integrating out the *𝜃* parameters. In this paper, we stick to the PJMLE for simplicity. Moreover, in recent studies, it has been demonstrated that PJMLE yields comparable estimates to the MMLE (Paolino, 2013; Chen, Li, & Zhang, 2019; Robitzsch, 2021). Given fixed GPCM parameters, we optimize the DIF parameters through coordinate descent at the second level, treating each *δ*_*if*_ for $i = 1,\ldots ,\mathbb {P}$ and *f* = 1,…,*m*_*f*_ as a unique coordinate. Details of the coordinate descent algorithm applied to Eq. 4 are provided in Appendix 2.

## The proposed method

### In-plus-out-of-questionnaire log likelihood with DIF

In instrument design, we are given an initial set of $\mathbb {P}$ items that, based on responses on a survey including all these items, we would like to reduce to a smaller set of items that make up the final questionnaire. We will refer to the set of included items as the included itemset, denoted ${\mathcal {S}_{\text {in}}}$, and to its complement as the excluded itemset, denoted ${\mathcal {S}_{\text {out}}} = \{1,\ldots ,\mathbb {P}\} \setminus {\mathcal {S}_{\text {in}}}$. In Wijayanto et al., (2021), we introduced a novel criterion called in-plus-out-of-questionnaire log likelihood (IPOQ-LL) for evaluating the quality of any split into ${\mathcal {S}_{\text {in}}}$ and ${\mathcal {S}_{\text {out}}}$ given the observed responses on the original survey. Following the same rationale, we here extend this criterion to also incorporate the possibility of item(s) with differential functioning.

For a given final questionnaire, only the items in the included itemset ${\mathcal {S}_{\text {in}}}$ can be used to estimate the subjects’ abilities *𝜃*. We propose to obtain these abilities, and at the same time the discrimination parameters, thresholds, and DIF parameters corresponding to the included items, by maximizing the penalized log likelihood in Eq. 4:

5 $$\begin{array}{@{}rcl@{}} \left\{\hat{\theta}_{{\mathcal{S}_{\text{in}}}},\hat{\beta}_{{\mathcal{S}_{\text{in}}}},\hat{\alpha}_{{\mathcal{S}_{\text{in}}}},\hat{\delta}_{{\mathcal{S}_{\text{in}}}}\right\} &=& \underset{\{\theta,\beta,\alpha,\delta\}}{\text{argmax}} L_{{\mathcal{S}_{\text{in}}}}(\theta,\beta,\alpha,\delta) - \lambda_{\theta} \sum\limits_{n=1}^{N} {\theta_{n}^{2}} \\&&- \lambda_{\text{in}} \sum\limits_{i \in {\mathcal{S}_{\text{in}}}} (\ln \alpha_{i})^{2} - \lambda_{\delta} \sum\limits_{i \in {\mathcal{S}_{\text{in}}}}\sum\limits_{f=1}^{m_{f}} |\delta_{i f}| . \end{array}$$

We refer to the log likelihood of these fitted parameters on the included itemset as the in-questionnaire log likelihood with DIF:

6 $$\text{IQ-LL-DIF}({\mathcal{S}_{\text{in}}}) = L_{{\mathcal{S}_{\text{in}}}}\left(\hat{\theta}_{{\mathcal{S}_{\text{in}}}},\hat{\beta}_{{\mathcal{S}_{\text{in}}}},\hat{\alpha}_{{\mathcal{S}_{\text{in}}}},\hat{\delta}_{{\mathcal{S}_{\text{in}}}}\right) .$$

This IQ-LL-DIF resembles standard test statistics in Rasch analysis (e.g., item fit statistics to the resolved DIF items) (Tennant et al.,, 2004, p I-40).

Next, although we may not need the excluded items to arrive at a reliable and valid scale, we would like the abilities estimated on ${\mathcal {S}_{\text {in}}}$ to properly represent the observed responses on ${\mathcal {S}_{\text {out}}}$ as well, if only because the original survey was designed to also include these items. We therefore fix the abilities $\hat {\theta }_{{\mathcal {S}_{\text {in}}}}$ and optimize the penalized log likelihood given the responses on the excluded items w.r.t. the thresholds, the discrimination parameters, and the DIF parameters:

7 $$\begin{array}{@{}rcl@{}} \left\{\hat{\beta}_{{\mathcal{S}_{\text{out}}}},\hat{\alpha}_{{\mathcal{S}_{\text{out}}}},\hat{\delta}_{{\mathcal{S}_{\text{out}}}}\right\} &=& \underset{\{\beta,\alpha\}}{\text{argmax}} L_{{\mathcal{S}_{\text{out}}}}(\hat{\theta}_{{\mathcal{S}_{\text{in}}}},\beta,\alpha) \\&&- \lambda_{\text{out}} \sum\limits_{i \in {\mathcal{S}_{\text{out}}}} (\ln \alpha_{i})^{2} - \lambda_{\delta} \sum\limits_{i \in {\mathcal{S}_{\text{out}}}}\sum\limits_{f=1}^{m_{f}} |\delta_{i f}| . \end{array}$$

We refer to

8 $$\text{OQ-LL-DIF}({\mathcal{S}_{\text{out}}}) = L_{{\mathcal{S}_{\text{out}}}}\left(\hat{\theta}_{{\mathcal{S}_{\text{in}}}},\hat{\beta}_{{\mathcal{S}_{\text{out}}}},\hat{\alpha}_{{\mathcal{S}_{\text{out}}}},\hat{\delta}_{{\mathcal{S}_{\text{out}}}}\right) .$$

as the out-of-questionnaire log likelihood with DIF. Our new criterion, the in-plus-out-of-questionnaire log likelihood with DIF (IPOQ-LL-DIF), is the total of both log likelihoods:

$$\text{IPOQ-LL-DIF}({\mathcal{S}_{\text{in}}},{\mathcal{S}_{\text{out}}}) = \text{IQ-LL-DIF}({\mathcal{S}_{\text{in}}}) + \text{OQ-LL-DIF}({\mathcal{S}_{\text{out}}}) .$$

Algorithm 1 outlines the procedure for computing the in-plus-out-of-questionnaire log likelihood with DIF given a subdivision of all items into the included itemset ${\mathcal {S}_{\text {in}}}$ and excluded itemset ${\mathcal {S}_{\text {out}}}$.

**Algorithm 1:**
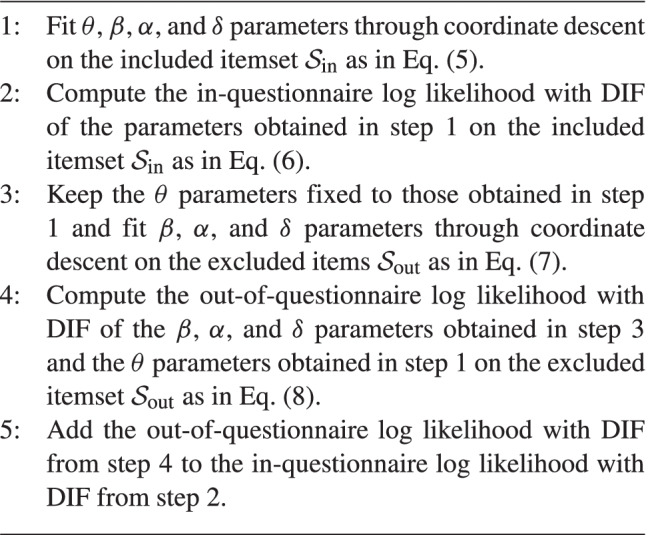
Pseudocode for computing the in-plus-out-of-questionnaire log likelihood with DIF for a particular included itemset ${\mathcal {S}_{\text {in}}}$ and excluded itemset ${\mathcal {S}_{\text {out}}}$.

In our earlier work (Wijayanto et al., 2021), we noticed that the outcome of our fitting procedure without the additional DIF parameters is relatively insensitive to the setting of the regularization parameters, as long as the regularization parameter *λ*_in_ of the *β* and *α* parameters for the included itemset is an order of magnitude larger than the regularization parameter *λ*_out_ for the excluded itemset. In this paper, we therefore stick to the same settings: *λ*_*𝜃*_ = 0.05, *λ*_in_ = 50, and *λ*_out_ = 1.

Whether or not non-zero DIF parameters are obtained, does depend on the precise setting of the regularization parameter *λ*_*δ*_: the larger *λ*_*δ*_, the fewer non-zero DIF parameters will remain. Unless specified otherwise, in this paper we set *λ*_*δ*_ = 10. With this setting, our procedure yields more or less the same DIF items in the three real-world datasets compared to those obtained with a manual analysis. An arguably more principled, but computationally much more intensive approach would be a cross-validation procedure for finding the optimal value of *λ*_*δ*_, as described in Appendix 2.

### Comparison with other approaches for DIF assessment

There are two main approaches for handling DIF items.

- Blending in with the Rasch analysis. In many practices of Rasch analysis, DIF detection is infused as an additional step in the estimation procedure (Rosato et al., 2016; Vaughan, 2018; 2019). Resolved DIF items are treated as any other items: if they fit the Rasch model well they are kept, otherwise they are removed. In accordance with our previous method (Wijayanto et al., 2021), our new method has a tendency to put predictive split-items in the included itemset: these items help to obtain a better estimate of the subjects’ ability not only in the included itemset, but also in the excluded itemset.

- Treating the DIF items separately. Andrich and Hagquist (2015) distinguish between ‘real’ and ‘artificial’ DIF items. A real DIF item is stable, independent of the inclusion or exclusion of other potential DIF items. An artificial DIF item, on the other hand, only becomes a DIF item by virtue of the presence of other (real) DIF items. Therefore, Andrich and Hagquist (2015) suggest to resolve the DIF items iteratively, starting with the largest effect, in an attempt to neutralize the effect of artificial DIF items. Our procedure also applies a thorough strategy to identify and resolve all potential DIF items. However, instead of doing this sequentially, we simultaneously estimate the DIF effects for all items that are still included. The Lasso (*L*1) penalty helps to distinguish between DIF and non-DIF items by nullifying the insignificant DIF effects.

### Itemset selection

In this paper, we introduce a single criterion, IPOQ-LL-DIF, to measure the quality of a final instrument by considering the differential functioning over items. With this criterion, we can in principle apply any optimization procedure to determine which items to keep in the included itemset ${\mathcal {S}_{\text {in}}}$ and which items to put in the excluded itemset ${\mathcal {S}_{\text {out}}}$. In our experiments, we consider the same optimization procedure as in our previous work (see Wijayanto et al., (2021) for details), i.e., stepwise selection. Stepwise selection alternates between backward elimination, which starts from the full set of items, and forward selection, which starts from the empty set. Starting from a full itemset, backward elimination will eliminate the item that corresponds to the highest IPOQ-LL-DIF. Forward selection gives the search procedure the ability to recover items later in the process.

## Experimental study

To evaluate our new method, we experiment on an artificial dataset and on three publicly available real-world datasets.

### Application to artificial data

This simulation aims to show that our semi-automated algorithm aligns with the standard Rasch analysis procedure for dealing with DIF, i.e., identifies, resolves, and removes split items that are relatively hard to predict. In this experiment, we consider an artificial dataset that consists of responses to 14 items from 490 subjects. The dataset is composed of two inhomogeneous subsets with six items (12 items in total) and two DIF items. To simulate the DIF effect, the subjects are split into two different groups of 245. Responses are generated independently from the generalized partial credit model for the polytomous case with *m*_*i*_ = 5 ordered categories.

The response in all inhomogeneous subsets are generated with the same person ability and item difficulty scores, *𝜃*_*n*_ = (0.02(*n* − 1) − 3) for $n = 1,\dots ,245$ and *β*_*ij*_ = ((*i* − 1) − (1.3 + 0.8(*j* − 1))) for $j = 1,\dots ,4$ and $i = 1,\dots ,6$, respectively. However, the discrimination parameters are varied among subsets, *α* = {0.2,2} for the first and second subsets, respectively. This should make responses on items in the first subset (item_1 − 6_) relatively hard to predict, and relatively easy to predict in the second subset (item_7 − 12_). To simulate the effect of DIF, responses of both DIF items are generated with different difficulty parameters for the two subgroups. We set the thresholds to *β* = {− 3.7,− 2.9,− 2.1,− 1.3} for the first and *β* = {1.3,2.1,2.9,3.7} for the second subgroup of the first DIF item (item_13_). As for the second DIF item (item_14_), we set *β* = {− 5.2,− 4.4,− 3.6,− 2.8} for the first subgroup, and *β* = {2.8,3.6,4.4,5.2} for the second. Furthermore, we choose item_13_ to be predictive and item_14_ to be hard-to-predict for both subgroups, by setting *α* = {0.8} and *α* = {0.1}, respectively.

**Fig. 1:**
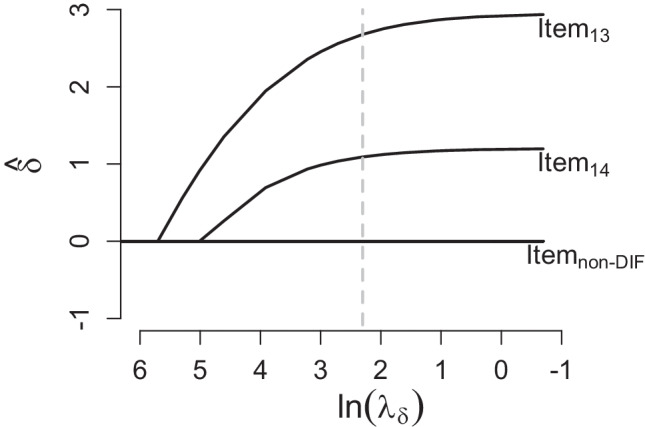
The estimated DIF parameters ($\hat {\delta }$) along $\log (\lambda _{\delta })$ for DIF and non-DIF items. The *dashed grey line* represents the value of *λ*_*δ*_ that is used in the estimation

For a given dataset containing DIF item(s), the PCM-DIF (see “Generalized partial credit model with DIF” section) can be applied to estimate the DIF parameters. Figure 1 shows that the PCM-DIF can identify the DIF items (item_13_ and item_14_) when the value of *λ*_*δ*_ is not too high. However, for a high value of *λ*_*δ*_, these DIF effects disappear and the PCM-DIF leads to the same estimated parameters as a standard PCM without DIF. The PCM-DIF correctly estimates the DIF parameters (*δ*) of all non-DIF items to equal zero for any value of *λ*_*δ*_.

**Fig. 2:**
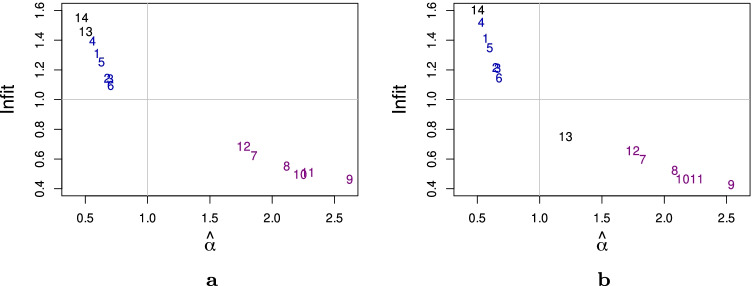
The estimated discrimination parameters ($\hat \alpha$) against the Infit statistics of (**a**) the PCM and (**b**) the PCM-DIF. Discrimination parameters (*α*) are estimated using (**a**) the GPCM and (**b**) the GPCM-DIF. The *horizontal line* represents the expected Infit value and vertical line represents the expected discrimination value

Infit is one of the item fit statistics that is commonly used to judge the goodness-of-fit of items to the Rasch model and to the PCM. In Fig. 2, we show that this statistic relates to the discriminative power of the items, represented by the discrimination parameter $\hat {\alpha }$. A hard-to-predict item with low discriminative power normally has a high Infit, which indicates misfit. On the contrary, an easy-to-predict item with high discriminative power normally has a low infit. Further, we also show that the misfitting item_13_ in Fig. 2a (estimated using the PCM) improves its Infit after considering the DIF effect (estimated using PCM-DIF). The PCM-DIF clearly models the responses of item_13_ better than the PCM. As expected, applying the PCM-DIF does not improve the Infit of item_14_, the hard-to-predict DIF item. For the non-DIF items, the PCM-DIF estimates are indistinguishable from the PCM estimates.

**Fig. 3:**
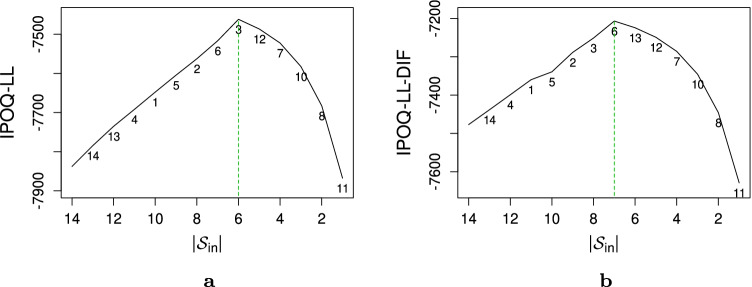
The highest IPOQ-LL and IPOQ-LL-DIF scores as a function of the number of included items $|{\mathcal {S}_{\text {in}}}|$ for the artificial dataset. **a** IPOQ-LL computed with GPCM. **b** IPOQ-LL-DIF computed with GPCM-DIF. The *numbers on the plot* shows in which order the items are removed

When DIF is present in particular items, standard Rasch analysis tends to detect and resolve these items. This step, together with expert awareness of the inspected items, is then followed by removing misfits, including those that are hard to predict even after splitting the DIF items. As shown in Fig. 3b, our semi-automated algorithm does the same for reasons explained in “Comparison with other approaches for DIF assessment” section: the IPOQ-LL-DIF score favors the DIF item that has a good fit after being split and puts the one that has a low discrimination parameter in the excluded set. The maximum of the IPOQ-LL-DIF score as a function of the number of items in the included set in this simulation is obtained when seven items are still included, including the resolved item_13_.

As a comparison, we also apply our previous criterion, the IPOQ-LL, to this dataset. Figure 3a shows that the IPOQ-LL detects item_13_ (the predictive item) as a hard-to-predict item since it cannot estimate the DIF effect. Consequently, when a potential DIF effect is ignored item_13_ will be put to the excluded itemset. As for item_14_, being designed as a hard-to-predict DIF item, both IPOQ-LL and IPOQ-LL-DIF agree to put it in the excluded itemset. This fact is also supported by Fig. 2 which shows that the Infit statistic of item_14_ does not improve even after the DIF effect has been identified.

### Application to real-world datasets

To validate our method on real-world data, we searched for datasets that satisfy the following criteria:

- The original dataset (survey with responses) is publicly available.
- A manual Rasch analysis has been applied to develop an instrument.
- According to the manual Rasch analysis, the initial survey contains differential item functioning.
- None of the authors of the current paper have been involved in the development of the instrument.
- The corresponding publication is not more than 5 years old.

We found three such datasets: the Osteopathy Clinical Teaching Questionnaire dataset (Vaughan, 2018), the Interdisciplinary Education Perception Scale dataset (Vaughan, 2019), and the Multiple Sclerosis Quality of Life Scale dataset (Rosato et al., 2016). To these three datasets, we applied our semi-automated procedure with the new criterion, the IPOQ-LL-DIF. For comparison, we also use the IPOQ-LL criterion.

#### The Osteopathy Clinical Teaching Questionnaire (OCTQ) dataset

The Osteopathy Clinical Teaching Questionnaire (OCTQ) is an instrument that was developed to assess the quality of the clinical educators (Vaughan, 2018). The original survey contains 30 items with five-point Likert scale and three global questions that have been answered by 399 participants. Vaughan (2018) performed a manual Rasch analysis and ended up with 12 items, ${\mathcal {S}_{\text {in}}} = \{2,5,7,9,10,12,15,16,18,20,23,30\}$, as the final instrument. We will refer to this set of 12 items as the OCTQ manual instrument.

**Fig. 4:**
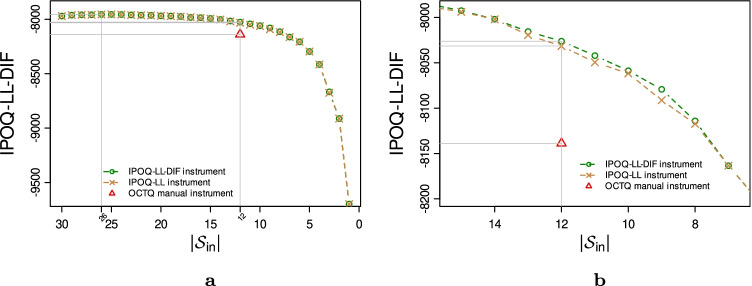
The highest IPOQ-LL-DIF scores as a function of the number of included items $|{\mathcal {S}_{\text {in}}}|$ when running the semi-automated procedure using the IPOQ-LL-DIF and IPOQ-LL criteria on the OCTQ dataset. Graph (**b**) zooms in on the number of included items close to those of the manual instrument

In the original survey, Vaughan (2018) identified some items with disordered thresholds, four items with DIF (item_14_, item_19_, item_27_, and item_28_), and 122 misfitting persons. After resolving the few items with disordered thresholds in the original survey (item_1_, item_9_, item_27_, and item_30_), we applied the semi-automated procedure for both criteria. Running the whole stepwise procedure leads to the result shown in Fig. 4. Both criteria agree that the maximum of the IPOQ-LL-DIF occurs when the same 26 items are still included. The vertical lines give the location of maximum scores, $|{\mathcal {S}_{\text {in}}}| = 26$ and $|{\mathcal {S}_{\text {in}}}| = 12$. The horizontal lines give the location of the corresponding scores and show the score differences among instruments. Figure 4b zooms in on the search result near $|{\mathcal {S}_{\text {in}}}| = 12$, the number of items in the OCTQ manual instrument.

For a fair and easy comparison with the manual instrument, we zoom in on the semi-automated instruments that are based on the same number of included items. We will refer to these as the IPOQ-LL-DIF (${\mathcal {S}_{\text {in}}} = \{2,3,5,7,10,12,14,16,18,22,26,30\}$) and IPOQ-LL (${\mathcal {S}_{\text {in}}} = \{2,3,5,7,10,12,14,16,17,22,26,30\}$) instruments, respectively. The semi-automated instruments only differ in one item: 17 versus 18. The overlap between the IPOQ-LL-DIF and the manual instrument is eight items, which can be considered large: the probability of having an overlap of eight or more items just by chance is smaller than 0.05. The overlap between the IPOQ-LL instrument and the manual instrument is seven items.

In the initial analysis, Vaughan (2018) suspected some items to be DIF items, i.e., item_27_, and item_28_ for institution, item_14_ for institution and educator gender, and item_19_ for student gender. As part of the Rasch analysis, Vaughan (2018) chose to remove all DIF items to ensure that the final version of OCTQ would be applicable to a range of teaching institutions and free from gender influence. Employing the IPOQ-LL-DIF criterion, our semi-automated procedure retains three DIF items, i.e., item_14_, item_22_, and item_26_.

To further illustrate the clinimetric quality of the three (i.e., manual, IPOQ-LL-DIF, and IPOQ-LL) instruments, we consider standard Rasch statistics such as goodness-of-fit, local independency, reliability, and unidimensionality. For comparison we also compute these statistics for 10,000 randomly drawn 12-item instruments. The statistics of the three instruments are all well within the acceptable range and, in this case, are better than most of the random 12-item instruments in local independency (see Appendix 1 for details). Furthermore, we compute the Cronbach–Mesbah curve (Fig. 13) to track how the instrument’s internal consistency changes over time (Mesbah, 2010). Despite the highest Cronbach’s *α* obtained after removing one item, the instrument with the highest IPOQ-LL-DIF score is also considered to have excellent internal consistency (*α* = 0.97).

**Fig. 5:**
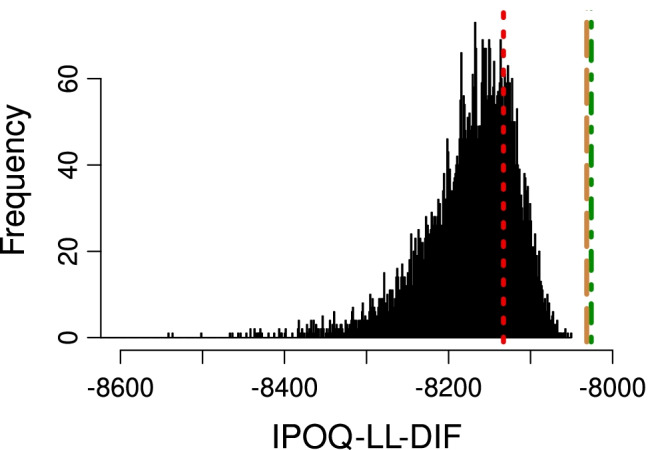
In-plus-out-questionnaire log likelihood with DIF (IPOQ-LL-DIF) values for the IPOQ-LL-DIF instrument (*green dotted-dashed line*), the IPOQ-LL instrument (*brown dashed line*), the manual instrument from Vaughan (2018) (*red dotted line*), and random 12-item instruments (*histogram*) on the OCTQ dataset

Figure 5 compares all instruments using our own IPOQ-LL-DIF criterion. By definition, the IPOQ-LL-DIF instrument is very well optimized for this criterion and slightly higher than the IPOQ-LL instrument. However, the manual instrument also does well and better than most of the randomly drawn 12-item instruments, which shows that as an extension of the IPOQ-LL criterion, the IPOQ-LL-DIF intrinsically captures many of the properties that a typical Rasch analysis cares about, including the presence of the DIF.

**Fig. 6:**
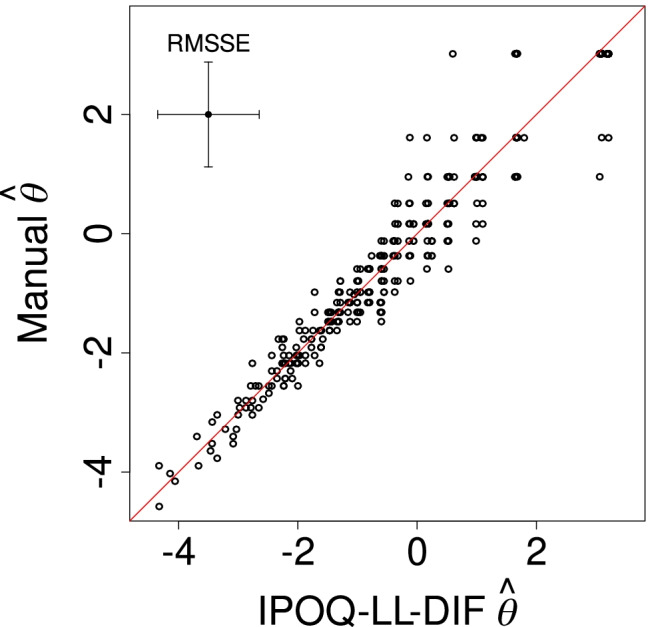
Estimated abilities for individual subjects based on the IPOQ-LL-DIF against those based on the manual instrument. The average root mean squared standard error for the estimates on both axes is visualized through the *error bars* at the top left

Considering the standard Rasch statistics, which are averages over all items and all subjects, we conclude that the manual and the semi-automated instruments are clinimetrically all very similar. We then also expect that the abilities estimated for individual subjects based on the manual and the IPOQ-LL-DIF instruments will be very much alike. Figure 6 plots these estimated ability parameters for the two instruments against each other. Indeed, the estimated ability parameters for the two instruments are highly correlated (*ρ* = 0.975), further showing that both instruments are very similar.

#### The interdisciplinary education perception scale (IEPS) dataset

The interdisciplinary education perception scale is an instrument to evaluate students’ professional perception in a particular program (Vaughan, 2019). The complete survey consists of 18 items of six-point Likert scale that are answered by 319 participants. Adopting the work of Leitch (2014) and Vaughan (2019) excluded item_12_ and item_17_ and applied a manual Rasch analysis to 16 items as the initial survey. During the analysis, Vaughan (2019) identified 51 misfitting persons, resolved four items with disordered thresholds (i.e., item_10_, item_13_, item_15_, and item_16_), removed eight items, and ended up with eight items as the final instrument. We will refer to this set of eight items, ${\mathcal {S}_{\text {in}}} = \{1,2,7,10,13,14,15,16\}$, as the IEPS manual instrument.

**Fig. 7:**
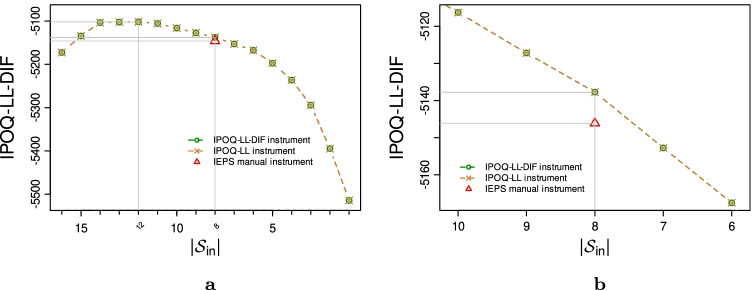
The highest IPOQ-LL-DIF scores obtained for each number of included items $|{\mathcal {S}_{\text {in}}}|$ when running the semi-automated procedure using both the IPOQ-LL-DIF and IPOQ-LL criteria on the IEPS dataset. **a** Of all available $|{\mathcal {S}_{\text {in}}}|$. **b** Zoomed in version from $|{\mathcal {S}_{\text {in}}}| = 6$ to $|{\mathcal {S}_{\text {in}}}| = 10$. The *horizontal grey lines* display the differences of the IPOQ-LL-DIF scores obtained by the three instruments, i.e., the manual, IPOQ-LL, and IPOQ-LL-DIF instruments

After resolving the four items with disordered thresholds, we applied our semi-automated procedure to the remaining 16-items IEPS responses. Running the whole stepwise procedure using the IPOQ-LL-DIF and IPOQ-LL criteria leads to the graph shown in Fig. 7. Both criteria agree that the maximum of the IPOQ-LL-DIF occurs when the same 12 items are still included. Using the same setup, the vertical lines give the location of the maximum scores, $|{\mathcal {S}_{\text {in}}}|= 12$ and $|{\mathcal {S}_{\text {in}}}|= 8$. The horizontal lines give the location of the corresponding scores and show the score differences among instruments.

For a fair and easy comparison with the manual instrument, we again zoom in on the semi-automated instruments that are based on the same number of included items as the manual instrument. We will refer to these as the IPOQ-LL-DIF and IPOQ-LL instruments, respectively, which happen to contain the exact same items (${\mathcal {S}_{\text {in}}} = \{1,2,4,5,7,13,15,16\}$). Figure 7b zooms in on the search result near $|{\mathcal {S}_{\text {in}}}| = 8$, the number of items in the IEPS manual instrument. The overlap between the IPOQ-LL-DIF and the manual instrument is 6 items. The probability of having an overlap of six or more items just by chance is 0.07.

Vaughan (2019) also reported the presence of three DIF items, i.e., item_6_ for year level, item_11_ for gender, and item_18_ for university. Vaughan (2019) chose to remove all DIF items in order to produce a questionnaire that is free of demographic influence. Our semi-automated procedure also led to the removal of these three potential DIF items, but for a different reason: they did not survive the selection procedure when optimizing the IPOQ-LL-DIF and IPOQ-LL.

**Fig. 8:**
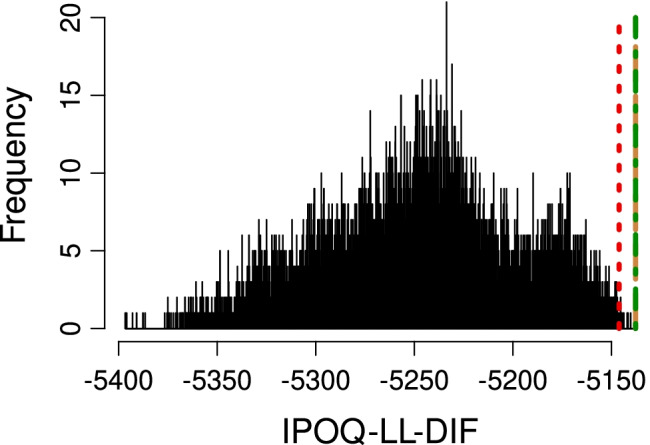
In-plus-out-questionnaire log likelihood with DIF (IPOQ-LL-DIF) values for the IPOQ-LL-DIF instrument (*green dotted-dashed line*), the IPOQ-LL instrument (*brown dashed line*), the manual instrument from Vaughan (2019) (*red dotted line*), and random eight-item instruments (*histogram*) on the IEPS dataset

**Fig. 9:**
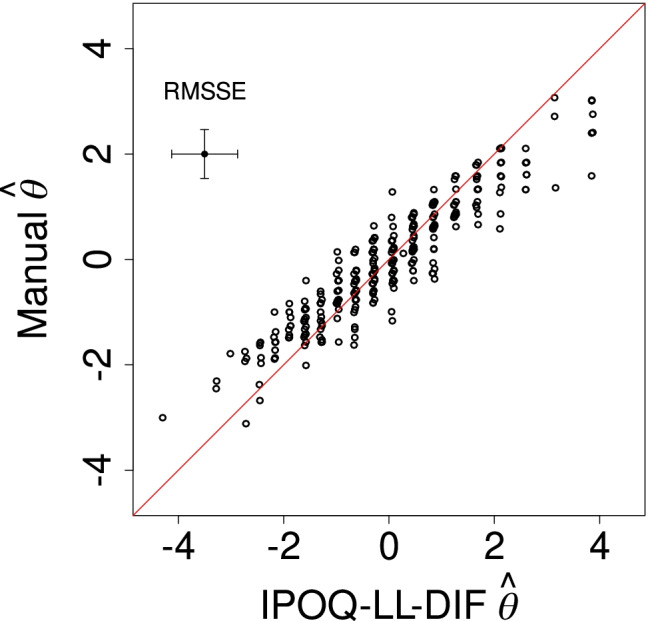
Estimated abilities for individual subjects based on the IPOQ-LL-DIF against those based on the manual instrument. The average root mean squared standard error for the estimates on both axes is visualized through the error bars at the top left

The figures in Appendix 1 show that the three (i.e., manual, IPOQ-LL-DIF, and IPOQ-LL) instruments are clearly better than most of the 10,000 randomly drawn eight-item instruments, especially on person separation reliability (PSR), local dependency, and unidimensionality. Furthermore, the statistics for these three instruments are clinimetrically very similar and all well within the acceptable range. Figure 8 shows that the manual instrument obtains an IPOQ-LL-DIF score that is only slightly smaller than the one for the semi-automated instruments. The estimated abilities of the manual and the semi-automated instruments indeed turn out to be very similar (*ρ* = 0.94) (see Fig. 9). Moreover, in Fig. 16, the Cronbach–Mesbah curve shows that the instrument that obtains the highest IPOQ-LL-DIF score also obtains the highest Cronbach’s *α*.

#### The multiple sclerosis quality of life (MSQOL) dataset

The multiple sclerosis quality of life questionnaire is an individual’s or a group’s perceived physical and mental health over time for people with multiple sclerosis (Rosato et al., 2016). The initial MSQOL survey consists of 54 items with different numbers of categories that was answered by 473 patients. The items are grouped into 12 multi-item and two single-item subscales. Rosato et al., (2016) applied separate manual Rasch analyses to 11 subscales, each originally containing at least three items. For two of these subscales, no items survived the analysis. We will refer to the remaining sets of nine subscales as the MSQOL manual instruments. They are listed in Table 3.

**Fig. 10:**
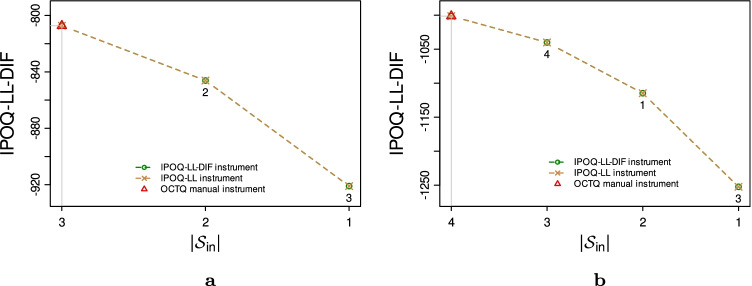
The highest IPOQ-LL-DIF scores obtained for each number of included items $|{\mathcal {S}_{\text {in}}}|$ when running the semi-automated procedure using either the IPOQ-LL-DIF or IPOQ-LL criterion to the MSQOL dataset on (**a**) subscale “Bodily Pain” and (**b**) subscale “Sexual Function”

For two subscales (“Bodily Pain” and “Sexual Function”), the manual Rasch analysis kept all items. Running our semi-automated stepwise procedure on these same subscales leads to the results shown in Fig. 10. It can be seen that the semi-automated procedure agrees to keep all items from both subscales: the maximum IPOQ-LL-DIF scores are obtained with all items still in the included set.

Next, we applied our semi-automated procedure on all 11 subscales, constraining the semi-automated instruments to end up with the same number of items as the corresponding manual instruments. For optimization with the IPOQ-LL-DIF and IPOQ-LL criteria we arrived at the exact same included item sets. We will refer to these as the MSQOL semi-automated instruments, also listed in Table 3. As can be seen, the semi-automated and manual instruments have 21 out of 27 items overlapping, which can be considered a lot: the probability of having an overlap of 21 or more items just by chance is smaller than 0.01.

Table 4 compares the psychometric quality of the manual and the semi-automated MSQOL instruments for all subscales. It can be seen that the standard Rasch statistics for both instruments are more or less the same.

Rosato et al., (2016) also reported the presence of eight DIF items, i.e., item_1_, item_8_, item_10_, item_23_, item_32_, item_36_, item_40_, and item_51_ and decided to remove these by hand. Our semi-automated procedure, however, retains two of these items, i.e., item_8_ and item_40_, albeit with corresponding DIF parameters $\hat {\delta }$ set to zero, i.e., without treating these as DIF items.

## Discussion and conclusions

In this work, we have successfully enhanced our semi-automated procedure to deal with DIF items. We extend our previous criterion, the so-called in-plus-out-of-questionnaire log likelihood (IPOQ-LL) to a new criterion named in-plus-out-of-questionnaire log likelihood with DIF (IPOQ-LL-DIF). The new criterion is based on the same ideas as the IPOQ-LL (Wijayanto et al., 2021): a good final instrument should reliably estimate people’s abilities. Although this ability estimate is fitted on the responses of the items in the final instrument, it should still represent the items that are left out.

The effectiveness of our extended procedure to yield clinimetrically similar results as the standard Rasch analyses relies on four essential ingredients. Two are passed down from the previous procedure, while the other two are new. The inherited ingredients are the flexible discrimination parameters and stronger regularization for this discrimination parameter on the included itemset compared to the excluded itemset (Wijayanto et al., 2021). The new ingredients are the DIF parameters together with a lasso penalty that distinguishes between the DIF and non-DIF items. We have shown that the new procedure also naturally incorporates essential aspects of Rasch analysis, where it tends to favor DIF items with a good Infit value and to exclude the ones without.

In our simulations, we have shown that DIF item(s) can indeed help obtain a better parameter estimate, in accordance with Andrich and Hagquist (2015). In a validation with real-world datasets our procedure yields similar instruments to the manual analyses. Not only do our instruments have comparable statistics, but they also contain similar (or even the same) items in the itemset when we constrain the number of included items to be the same as the number of included items in the manual instrument. With reasonable settings for the regularization parameters, our procedure tends to be somewhat more conservative in that it typically prefers to keep more items than the manual instrument.

In our experiments on real-world data, the IPOQ-LL-DIF and the IPOQ-LL criterion lead to very similar, often even the same instruments. Even though our procedure does detect and include DIF items, properly modeling this DIF effect has a relatively small effect on the selection of other items. However, this does depend on the setting of the regularization parameter *λ*_*δ*_. In our main experiments, we chose *λ*_*δ*_ = 10, which is relatively small, to arrive at more or less the same DIF items as in the manual analysis. An alternative approach would be to optimize *λ*_*δ*_ in a cross-validation procedure (see Appendix 2). Applying this cross-validation procedure to the real-world data, we obtain a much larger *λ*_*δ*_. With this stronger penalty, our procedure no longer finds any DIF items (see Fig. 17).

**Fig. 11:**
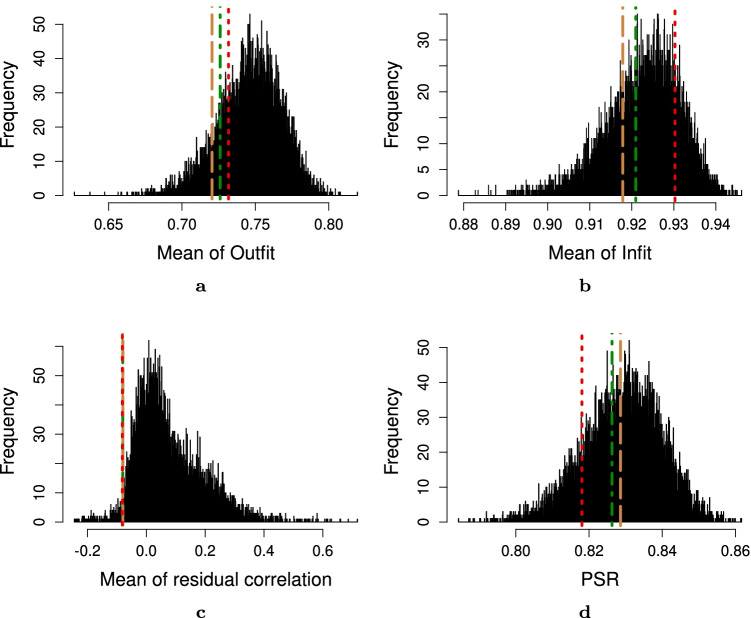
Statistics of the original 12-item instrument from Vaughan (2018) obtained through manual Rasch analysis (*red dotted line*), the optimal 12-item instrument according to the IPOQ-LL-DIF criterion (*green dotted-dashed line*), IPOQ-LL criterion (*brown dashed line*), and 10,000 random 12-item instruments (*histogram*) on the OCTQ dataset. **a** Mean of Outfit MnSq. **b** Mean of Infit MnSq. **c** Mean of residual correlation. **d** Person separation reliability (PSR)

To summarize, in our real-world experiments the manual, IPOQ-LL-DIF, and IPOQ-LL instruments are largely comparable and have clinimetrically similar qualities. Compared to randomly generated instruments, they all score well on standard Rasch statistics (see Figs. 11 through 15).

In this paper, we have assumed that the DIF groups are specified beforehand. For binary types of information (e.g., gender), these groups are naturally defined. For continuous type information (e.g., age), our procedure can be easily extended with a recursive partitioning method based on the IPOQ-LL-DIF to find the optimal groups, along the lines of some recent methods for detecting DIF (Strobl, Kopf, & Zeileis, 2015; Tutz & Berger, 2016; Komboz et al., 2018).

**Fig. 12:**
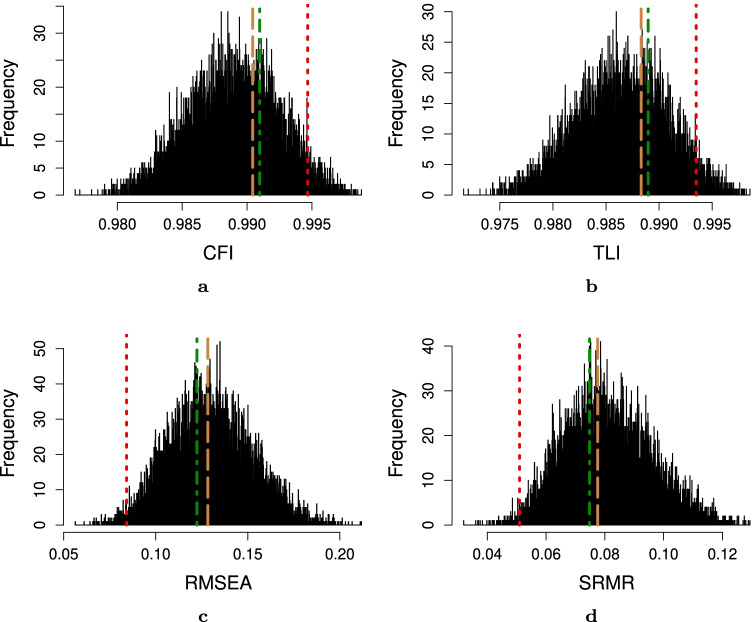
Unidimensionality test indices for the original 12-item instrument from Vaughan (2018) obtained through confirmatory factor analysis (*red dotted line*), the optimal 12-item instrument according to the IPOQ-LL-DIF criterion (*green dotted-dashed line*), IPOQ-LL criterion (*brown dashed line*), and 10,000 random 12-item instruments (*histogram*) on the osteopathy clinical teaching questionnaire dataset. **a** Comparative fit index (CFI). **b** Tucker–Lewis index (TLI). **c** Root mean-square error of approximation (RMSEA). **d** Standardized root mean squared residual (SRMR)

**Fig. 13:**
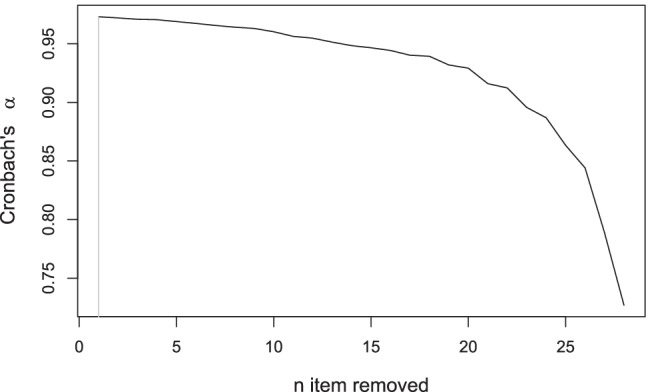
The Cronbach–Mesbah curve shows the changes in internal consistency of the OCTQ instrument when an item is removed one at a time. The order of item removals is based on the semi-automated process. The *vertical line* shows the highest value of Cronbach’s *α*

**Fig. 14:**
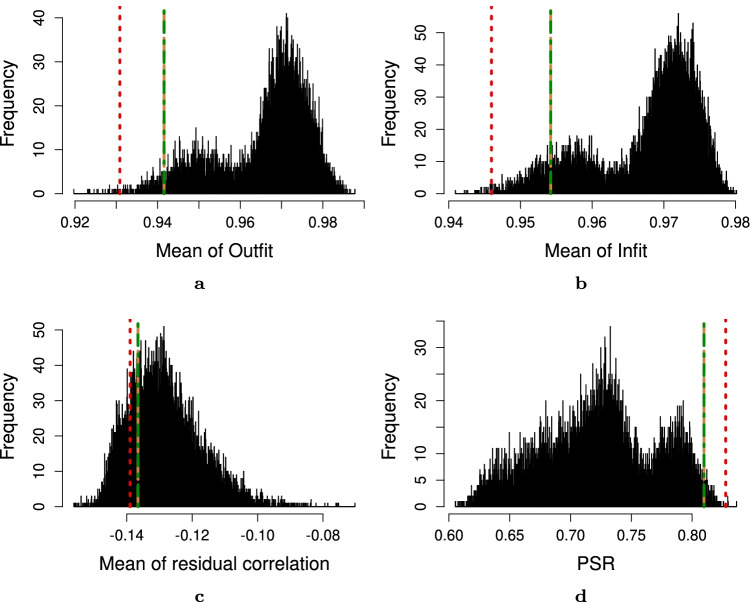
Statistics of the original 12-item instrument from Vaughan (2019) obtained through manual Rasch analysis (*red dotted line*), the optimal eight-item instrument according to the IPOQ-LL-DIF criterion (*green dotted-dashed line)*, IPOQ-LL criterion (*brown dashed line*), and 10,000 random eight-item instruments (*histogram*) on the interdisciplinary education perception scale dataset. **a** Mean of Outfit MnSq. **b** Mean of Infit MnSq. **c** Mean of residual correlation. **d** Person separation reliability (PSR)

**Fig. 15:**
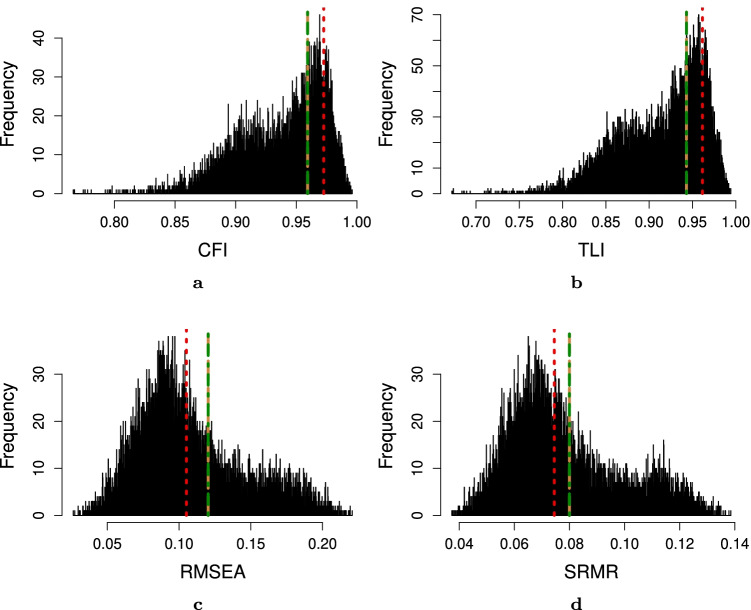
Unidimensionality test indices for the original eight-item instrument from Vaughan (2019) obtained through confirmatory factor analysis (*red dotted line*), the optimal eight-item instrument according to the IPOQ-LL-DIF criterion (*green dotted-dashed line*), IPOQ-LL criterion (*brown dashed line*), and 10,000 random eight-item instruments (*histogram*) on the interdisciplinary education perception scale dataset. **a** Comparative fit index (CFI). **b** Tucker–Lewis index (TLI). **c** Root mean-square error of approximation (RMSEA). **d** Standardized root mean squared residual (SRMR)

**Fig. 16:**
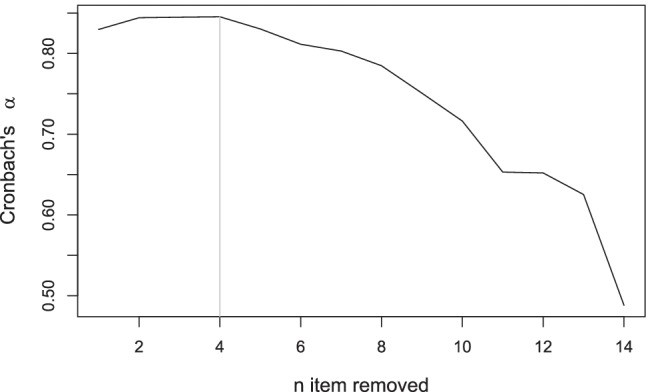
The Cronbach–Mesbah curve shows the changes in internal consistency of the IEPS instrument when an item is removed one at a time. The order of item removals is based on the semi-automated process. The *vertical line* shows the highest value of Cronbach’s *α*

**Fig. 17:**
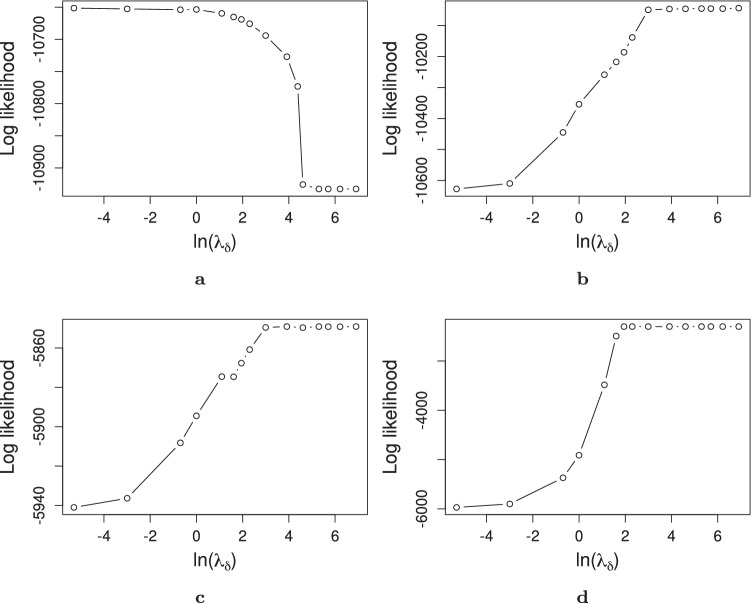
Results of cross-validation of four datasets using different values of *λ*_*δ*_. **a** The artificial dataset. **b** The OCTQ dataset. **c** The IEPS dataset. **d** The first dimension of the MSQOL dataset

Even though our method has the advantage to develop a valid, reliable, and robust instrument in a less time-consuming and more objective manner from a decent original survey, we are aware that this method lacks substantive human knowledge in the process. Knowing this, we are careful to frame our procedure as semi-automated rather than fully automated, which always welcomes the application of experts’ knowledge, e.g., through pre- and post-analysis (Fig. 12, 13, 14, 15 and 16).

## Open practices statements

The data that support the findings of this study are openly available in figshare at 10.6084/m9.figshare.c.3816553.v1, within the publication of: Vaughan (2018)

figshare at https://figshare.com/s/710483e7d6f574597518, within the publication of: Vaughan (2019)

figshare at 10.1371/journal.pone.0153466.s003, within the publication of: Rosato et al., (2016).

The R package containing the algorithm and results reported in this paper can be found at https://github.com/fwijayanto/autoRasch.

## Figures and Tables

**Algorithm 2:**
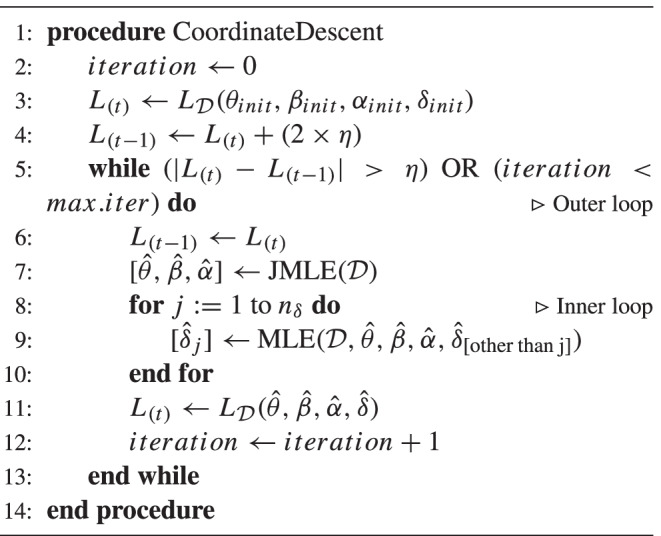
Pseudocode for two-level coordinate descent.

**Algorithm 3:**
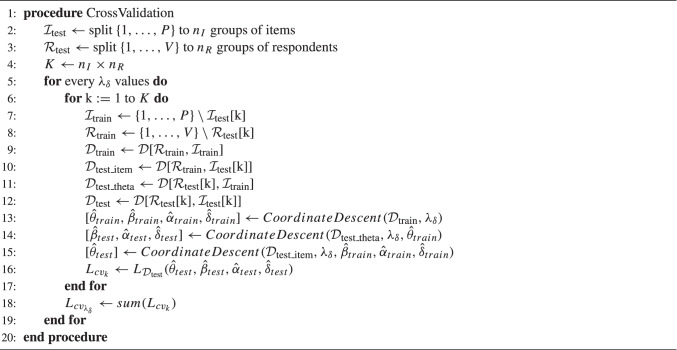
Pseudocode for k-fold cross-validation.

**Fig. 18:**
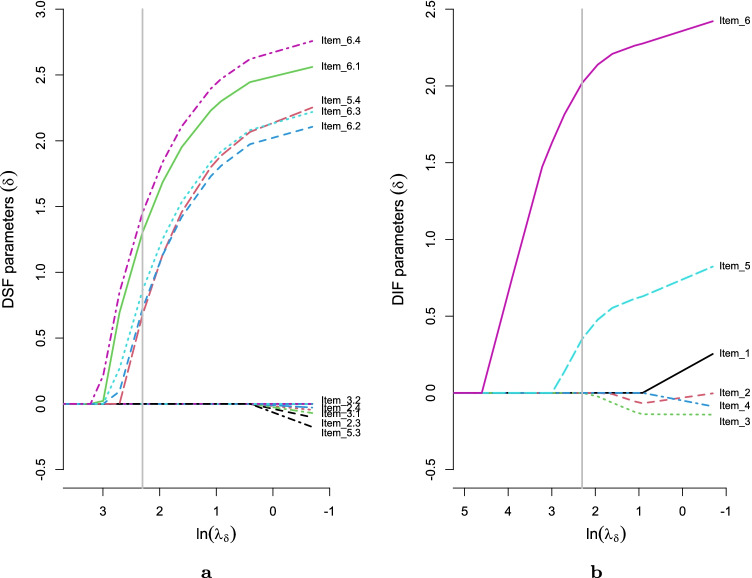
The estimated DSF and DIF parameters ($\hat {\delta }$) along $\log (\lambda _{\delta })$ for DSF and non-DSF items. The *vertical grey line* represents the value of *λ*_*δ*_ that is used in the estimation

## Appendix: 1

This section describes the results of the standard Rasch statistics applied to the OCTQ, IEPS, and MSQOL datasets. The comparison considers manual, IPOQ-LL-DIF, IPOQ-LL, and randomly drawn instruments. We examine goodness-of-fit, local dependency, unidimensionality, and reliability. Unlike unidimensionality and reliability, which assess the performance of an instrument, goodness-of-fit (i.e., Outfit and Infit) and local dependency (i.e., residual correlation) evaluate individual item performance. For Outfit and Infit, acceptable values are 0.6–1.4 for all items in a Likert type survey (Bond and Fox, 2015). As for residual correlations, a commonly used threshold is 0.2 or 0.3. However, higher thresholds (e.g., 0.5 and 0.7) are also frequently used (Christensen, Makransky, & Horton, 2017). For comparing the instruments’ performance, we take the mean of these statistics over all items.

Confirmatory factor analysis (CFA) is utilized to investigate the unidimensionality and the person separation reliability (PSR) measures the reliability of the estimated parameters. The combination of comparative fit index (CFI), Tucker–Lewis index (TLI), root mean square of approximation (RMSEA), and standardized root mean square residual (SRMR) is often used to describe the unidimensionality with particular thresholds, e.g., CFI > 0.95, TLI > 0.95, RMSEA < 0.06, and SRMR < 0.08 (Hu & Bentler, 1999). As for reliability, PSR ≥ 0.7 is considered acceptable, PSR ≥ 0.8 means good reliability, and PSR ≥ 0.9 is excellent (Duncan, Bode, Lai, & Perera, 2003) (Tables 1, 2, 3 and 4).

### A.1 OCTQ dataset analysis results

**Table 1:** The results of Rasch standard statistics on the OCTQ dataset for the three instruments, i.e., the manual, IPOQ-LL-DIF, and IPOQ-LL instruments

No.	Statistics	Manual	IPOQ-LL-DIF	IPOQ-LL
		items	items	items
1	Outfit	0.61 - 0.97	0.53 - 1.01	0.53 - 1.03
		(mean = 0.732)	(mean = 0.726)	(mean = 0.721)
2	Infit	0.75 - 1.13	0.73 - 1.18	0.72 - 1.21
		(mean = 0.930)	(mean = 0.921)	(mean = 0.918)
3	Residual correlation	mean = -0.08	mean = -0.08	mean = -0.08
4	PSR	0.818	0.826	0.829
5	Unidimensionality	CFI = 0.995,	CFI = 0.991	CFI = 0.990
		TLI = 0.993,	TLI = 0.989	TLI = 0.988
		RMSEA = 0.084,	RMSEA = 0.122	RMSEA = 0.128
		SRMR = 0.051	SRMR = 0.075	SRMR = 0.078

### A.2 IEPS dataset analysis results

**Table 2:** The result of Rasch standard statistics on the IEPS dataset for the three instruments, i.e., the manual, IPOQ-LL-DIF, and IPOQ-LL instruments

No.	Statistics	Manual	IPOQ-LL-DIF	IPOQ-LL
		items	items	items
1	Outfit	0.82 - 1.06	0.83 - 1.21	0.83 - 1.21
		(mean = 0.931)	(mean = 0.94)	(mean = 0.94)
2	Infit	0.86 - 1.03	0.85 - 1.19	0.85 - 1.19
		(mean = 0.946)	(mean = 0.954)	(mean = 0.954)
3	Residual correlation	mean = -0.139	mean = -0.137	mean = -0.137
4	PSR	0.83	0.81	0.81
5	Unidimensionality	CFI = 0.97,	CFI = 0.96	CFI = 0.96
		TLI = 0.96,	TLI = 0.94	TLI = 0.94
		RMSEA = 0.11,	RMSEA = 0.12	RMSEA = 0.12
		SRMR = 0.07	SRMR = 0.08	SRMR = 0.08

**Table 3:** Items included in the two instruments, i.e., the MSQOL manual, and IPOQ-LL-DIF, for 11 subscales

No.	Subscale	Manual items	IPOQ-LL-DIF items
1	Physical Function	4,5,6,7,9,11	3,5,7,8,9,11
2	Role Limitations — Physical	−	−
3	Role Limitations — Emotional	−	−
4	Bodily Pain	21,22,52	21,22,52
5	Emotional Wellbeing	25,26,30	25,26,28
6	Energy	27,29,31	27,29,31
7	Health Perceptions	35	34
8	Social Function	33	20
9	Cognitive Function	42,43,44	42,43,44
10	Health Distress	38,39,41	38,39,40
11	Sexual Function	46,47,48,49	46,47,48,49

The numbers of the items in the IPOQ-LL-DIF instruments are constrained to the number of the items in the corresponding manual instruments

**Table 4:** Standard Rasch statistics for the manual and IPOQ-LL-DIF MSQOL instruments. We only report those subscales for which the two instruments differ, i.e., “Physical Function”, “Emotional Wellbeing”, “Health Perceptions”, “Social Function”, and “Health Distress”

No.	Statistics	Manual items	IPOQ-LL-DIF items
Physical Function			
1	Outfit	0.32 - 0.46	0.30 - 0.65
		mean = 0.4	mean = 0.44
2	Infit	0.54 - 0.69	0.55 - 0.71
		mean = 0.62	mean = 0.64
3	PSR	0.78	0.80
4	Residual Correlation	no *r* > 0.3	no *r* > 0.3
		mean = -0.11	mean = -0.12
5	CFA	CFI = 1	CFI = 1
		TLI = 1	TLI = 1
		RMSEA = 0.82	RMSEA = 0.3
		SRMR = 0.02	SRMR = 0.014
6	IPOQ-LL-DIF	− 2010.68	− 1966.41
Emotional Wellbeing			
1	Outfit	0.65 - 0.72	0.50 - 0.81
		mean = 0.67	mean = 0.61
2	Infit	0.67 - 0.73	0.51 - 0.85
		mean = 0.69	mean = 0.63
3	PSR	0.79	0.82
4	Residual Correlation	no *ρ* > 0.3	no *ρ* > 0.3
		mean = -0.45	mean = -0.44
5	CFA	CFI = 1	CFI = 1
		TLI = 1	TLI = 1
		RMSEA = 0	RMSEA = 0
		SRMR = 0	SRMR = 0
6	IPOQ-LL-DIF	− 2177.78	− 2123.44
Health Perceptions			
1	Outfit	0.16	0.15
2	Infit	0.12	0.13
3	IPOQ-LL-DIF	− 2641.10	− 2559.88
Social Function			
1	Outfit	0.14	0.14
2	Infit	0.08	0.09
4	IPOQ-LL-DIF	− 1291.04	− 1281.129
Health Distress			
1	Outfit	0.41 - 0.49	0.45 - 0.57
		mean = 0.46	mean = 0.49
2	Infit	0.45 - 0.53	0.47 - 0.61
		mean = 0.50	mean = 0.53
3	PSR	0.83	0.84
4	Residual Correlation	no *ρ* > 0.3	no *ρ* > 0.3
		mean = -0.36	mean = -0.38
5	CFA	CFI = 1	CFI = 1
		TLI = 1	TLI = 1
		RMSEA = 0	RMSEA = 0
		SRMR = 0	SRMR = 0
6	IPOQ-LL-DIF	− 1460.23	− 1456.97

### A.3 MSQOL dataset analysis results

This section describes the analysis results applied to the 11 subscales of the MSQOL dataset. Table 3 shows the final instruments obtained by the manual Rasch analysis and the semi-automated procedure for each subscale. For all subscales, the IPOQ-LL instruments contain the same items as the IPOQ-LL-DIF instruments. Table 4 reports the results of the commonly used standard Rasch statistics for subscales of which the manual and the semi-automated instruments show some differences in item preferences.

## Appendix: 2

### B.1 Coordinate descent

Due to the addition of an *L*1 (lasso) penalty in the current model, joint maximum likelihood estimation (JMLE) requires a more involved procedure. We propose to apply a two-level coordinate descent as described in Algorithm 2. In the outer loop, we consider two sets of parameters. Our first coordinate contains all GPCM parameters (i.e., *𝜃*, *β*, and *α*), our second coordinate the DIF parameters (*δ*). Fixing the DIF parameters, we can optimize for the GPCM parameters. Given fixed GPCM parameters, we then have an inner loop in which we consider the DIF parameters (*δ*) as separate coordinates and solve them sequentially.

### B.2 Parameter Setting

To tune the *λ*_*δ*_ to its optimal value, some methods are available. These methods include model selection criteria, e.g., Akaike information criterion (AIC) (Akaike, 1974) and Bayesian information criterion (BIC) (Schwarz, 1978), as well as *k*-fold cross-validation (Schauberger and Mair, 2020). In this work, we implement the *k*-fold cross-validation procedure described in Algorithm 3. The items and subjects are split into several groups whose combinations constitute small blocks of the dataset. During cross-validation, we use one block as a test set and the other as a training set for every iteration.

We apply Algorithm 3 for cross-validation and the results are shown in Fig. 17. In Fig. 17a, we see that for the artificial dataset it is better to give no penalty to the DIF parameters (*δ*). In contrast, Fig. 17b, c, and d on the real-world datasets suggest giving a large penalty to the DIF parameters (*δ*), which makes the DIF model equivalent to a non-DIF model. This suggests that the responses on these real-world datasets are not significantly different between the different DIF groups.

### B.3 Differential step functioning

Penfield (2007) introduces the concept of step functions (corresponding to the thresholds in the PCM) that describe at which particular level of ability a subject steps (or advances) from one score level to a higher level. For a given observed response *x*_*n**i*_ ∈{0, 1,…,*m*_*i*_}, there will be *m*_*i*_ step functions. In the dichotomous case, with *m*_*i*_ = 1, any inconsistency between groups in the probability to score high can be modelled as a DIF in item level. In the polytomous case, with *m*_*i*_ > 1, however, this inconsistency may vary for every step function and is then called differential step functioning (DSF). To link DSF and DIF, Penfield, Gattamorta, and Childs (2009) explains the DIF effect as the aggregated DSF effect across the *m*_*i*_ steps. To implement DSF, we need to generalize Eq. 1 to

A1 $$\begin{array}{@{}rcl@{}} &&P(X_{ni}=x|\theta,\beta,\alpha,\delta) \\&=& \frac{\displaystyle \exp \left[\alpha_{i} \sum\limits_{j=1}^{x} \left(\theta_{n}-\left(\beta_{ij}-\sum\limits_{f=1}^{m_{f}}\delta_{jif}\kappa_{nf}\right)\right)\right]}{\displaystyle 1+\sum\limits_{k=1}^{m_{i}}\exp\left[\alpha_{i} \sum\limits_{j=1}^{k} \left(\theta_{n}-\left(\beta_{ij}-\sum\limits_{f=1}^{m_{f}}\delta_{jif}\kappa_{nf}\right)\right)\right]} , \end{array}$$

where we only had to change DIF parameter *δ*_*i**f*_ into the DSF parameter *δ*_*j**i**f*_, which now also depends on the category *j*.

As an experiment with the DSF model, we make use of an artificial dataset comprised of responses to six items from 400 subjects. The items composed of four non-DSF items and two DSF items. To simulate the DSF effect, the subjects are split into two different groups of 200. Responses are generated independently from the partial credit model (*α* = 1) for the polytomous case with five ordered categories (*m*_*i*_ = 4).

The response to all items are generated with the same person ability scores, *𝜃*_*n*_ = (0.05(*n* − 1) − 5) for $n = 1,\dots ,200$. The difficulty scores of the non-DSF items are set to *β*_*i**j*_ = (2.67(*i* − 1) − (2.8 + 0.8(*j* − 1))) for $j = 1,\dots ,4$ and $i = 1,\dots ,4$. To simulate the DSF effects, responses of DSF item are generated with difficulty parameters for the two subgroups. We set the thresholds to *β* = {− 4.2,− 3.4,− 2.6,− 1.8} for the first and *β* = {− 4.2,− 3.4,− 2.6, 0.6} for the second subgroup of the first DSF item (item_5_). As for the second DSF item (item_6_), we set *β* = {− 2.7,− 1.9,− 1.1,− 0.3} for the first subgroup, and *β* = {0.3, 1.1, 1.9, 2.7} for the second.

Figure 18 shows the paths of the estimated DSF (a) and DIF (b) parameters for various values of *λ*_*δ*_. These figures show that the GPCM-DSF model could satisfactorily identify the differential functioning at step 4 of item_5_ and all steps in item_6_. Despite item_5_ having a large DSF effect at step 4, the other steps have zero. As a result, as discussed by Penfield et al., (2009), the DIF effect of item_5_ as an aggregation of all DSF effects will be small.

## References

[CR1] Akaike H (1974). A new look at the statistical model identification. IEEE Transactions on Automatic Control.

[CR2] Andrich D, Hagquist C (2015). Real and artificial differential item functioning in polytomous items. Educational and Psychological Measurement.

[CR3] Bollmann S, Berger M, Tutz G (2018). Item-focused trees for the detection of differential item functioning in partial credit models. Educational and Psychological Measurement.

[CR4] Bond TG, Fox CM (2015). Applying the Rasch model: Fundamental measurement in the human sciences.

[CR5] Borsboom, D. (2006). When does measurement invariance matter? *Medical Care, 44*(11 Suppl 3). 10.1097/01.mlr.0000245143.08679.cc 17060825

[CR6] Chen, W.-H., & Revicki, D. (2014). Differential item functioning (DIF). In A.C. Michalos (Ed.) *Encyclopedia of quality of life and well-being research* (pp. 1611–1614). Dordrecht: Springer Netherlands.

[CR7] Chen Y, Li X, Zhang S (2019). Joint maximum likelihood estimation for high-dimensional exploratory item factor analysis. Psychometrika.

[CR8] Choi SW, Gibbons LE, Crane PK (2011). lordif: An R package for detecting differential item functioning using iterative hybrid ordinal logistic regression/item response theory and Monte Carlo simulations. Journal of Statistical Software.

[CR9] Christensen KB, Makransky G, Horton M (2017). Critical values for Yen’s Q3: Identification of local dependence in the Rasch model using residual correlations. Applied Psychological Measurement.

[CR10] Duncan PW, Bode RK, Lai SM, Perera S (2003). Rasch analysis of a new stroke-specific outcome scale: The stroke impact scale. Archives of Physical Medicine and Rehabilitation.

[CR11] Friedman J, Hastie T, Höfling H, Tibshirani R (2007). Pathwise coordinate optimization. The Annals of Applied Statistics.

[CR12] Hagquist C, Andrich D (2017). Recent advances in analysis of differential item functioning in health research using the Rasch model. Health and Quality of Life Outcomes.

[CR13] Holland PW, Thayer DT (1986). Differential item functioning and the Mantel-Haenszel procedure. ETS Research Report Series.

[CR14] Holland, P.W., & Wainer, H. (1993). *Differential item functioning*. Lawrence Erlbaum Associates, Inc.

[CR15] Hu L-T, Bentler PM (1999). Cutoff criteria for fit indexes in covariance structure analysis: Conventional criteria versus new alternatives. Structural Equation Modeling.

[CR16] Jeon M, Rijmen F (2016). A modular approach for item response theory modeling with the R package flirt. Behavior Research Methods.

[CR17] Komboz B, Strobl C, Zeileis A (2018). Tree-based global model tests for polytomous Rasch models. Educational and Psychological Measurement.

[CR18] Kopf J, Zeileis A, Strobl C (2015). A framework for anchor methods and an iterative forward approach for DIF detection. Applied Psychological Measurement.

[CR19] Kreiner S, Christensen KB (2011). Item screening in graphical loglinear Rasch models. Psychometrika.

[CR20] Leitch J (2014). Exploring psychometric properties of the interdisciplinary education perception scale in health graduate students. Journal of Interprofessional Care.

[CR21] Lord, F.M., & Novick, M.R (1968). Statistical theories of mental test scores, Addison-Wesley, Reading.

[CR22] Magis D, Facon B (2013). Item purification does not always improve DIF detection. Educational and Psychological Measurement.

[CR23] Magis, D., & Facon, B. (2014). deltaPlotR : An R package for differential item functioning analysis with Angoff’s delta plot. *Journal of Statistical Software, 59*(Code Snippet 1).

[CR24] Masters GN (1982). A Rasch model for partial credit scoring. Psychometrika.

[CR25] Mesbah, M. (2010). Statistical quality of life. In N. Balakrishnan (Ed.) *Methods and applications of statistics in the life and health sciences* (pp. 839–864): Wiley.

[CR26] Muraki, E. (1992). A generalized partial credit model: Application of an EM algorithm. *Applied Psychological Measurement, 16*(2).

[CR27] Paolino, J.-P. (2013). *Penalized joint maximum likelihood estimation applied to two parameter logistic item response models*. PhD thesis, Columbia University.

[CR28] Penfield RD (2007). Assessing differential step functioning in polytomous items using a common odds ratio. Journal of Educational Measurement.

[CR29] Penfield RD, Gattamorta K, Childs RA (2009). An NCME instructional module on using differential step functioning to refine the analysis of DIF in polytomous items. Educational Measurement: Issues and Practice.

[CR30] Rasch, G. (1960). *Studies in mathematical psychology: I. Probabilistic models for some intelligence and attainment tests*. Nielsen & Lydiche.

[CR31] Robitzsch A (2021). A comprehensive simulation study of estimation methods for the Rasch model. Stats.

[CR32] Rosato R, Testa S, Bertolotto A, Confalonieri P, Patti F, Lugaresi A, Grasso MG, Toscano A, Giordano A, Solari A (2016). Development of a short version of MSQOL- 54 using factor analysis and item response theory. PLoS ONE.

[CR33] Schauberger G, Mair P (2020). A regularization approach for the detection of differential item functioning in generalized partial credit models. Behavior Research Methods.

[CR34] Schneider, L., Strobl, C., Zeileis, A., & Debelak, R (2021). An R toolbox for score-based measurement invariance tests in IRT models. *Behavior Research Methods*. 10.3758/s13428-021-01689-0 PMC9579078 34918222

[CR35] Schwarz G (1978). Estimating the dimension of a model. Annals of Statistics.

[CR36] Strobl C, Kopf J, Zeileis A (2015). Rasch trees: A new method for detecting differential item functioning in the Rasch model. Psychometrika.

[CR37] Swaminathan H, Rogers HJ (1990). Detecting differential item functioning using logistic regression procedures. Journal of Educational Measurement.

[CR38] Tennant A, Penta M, Tesio L, Grimby G, Thonnard J-L, Slade A, Lawton G, Simone A, Carter J, Lundgren-Nilsson Å, Tripolski M, Ring H, Biering-Sørensen F (2004). Assessing and adjusting for cross-cultural validity of impairment and activity limitation scales through differential item functioning within the framework of the Rasch model. Medical Care.

[CR39] Tutz G, Berger M (2016). Item-focussed trees for the identification of items in differential item functioning. Psychometrika.

[CR40] Tutz G, Schauberger G (2015). A penalty approach to differential item functioning in Rasch models. Psychometrika.

[CR41] Vaughan B (2018). Exploring the measurement properties of the osteopathy clinical teaching questionnaire using Rasch analysis. Chiropractic and Manual Therapies.

[CR42] Vaughan B (2019). Measurement properties of the Interdisciplinary Education Perception Scale in an Australian allied health student cohort. Health Professions Education.

[CR43] Wijayanto F, Mul K, Groot P, van Engelen BG, Heskes T (2021). Semi-automated Rasch analysis using in-plus-out-of-questionnaire log likelihood. British Journal of Mathematical and Statistical Psychology.

[CR44] www.rasch.org (2014). Rasch measurement analysis software directory.

